# Assessment methods in medical specialist assessments in the DACH region – overview, critical examination and recommendations for further development

**DOI:** 10.3205/zma001286

**Published:** 2019-11-15

**Authors:** Nils Thiessen, Martin R. Fischer, Sören Huwendiek

**Affiliations:** 1 EDU - a degree smarter, Digital Education Holdings Ltd., Kalkara, Republic of Malta; 2 LMU München, Klinikum der Universität München, Institut für Didaktik und Ausbildungsforschung in der Medizin, München, Germany; 3 Universität Bern, Institut für Medizinische Lehre, Abteilung für Assessment und Evaluation, Bern, Switzerland

**Keywords:** medical specialist assessment, DACH region, cognitive, practical and communicative competencies

## Abstract

**Introduction: **Specialist medical assessments fulfil the task of ensuring that physicians have the clinical competence to independently represent their field and provide the best possible care to patients, taking into account the current state of knowledge. To date, there are no comprehensive reports on the status of specialist assessments in the German-speaking countries (DACH). For that reason, the assessment methods used in the DACH region are compiled and critically evaluated in this article, and recommendations for further development are described.

**Methods: **The websites of the following institutions were searched for information regarding testing methods used and the organisation of specialist examinations:

1. Homepage of the Swiss Institute for Medical Continuing Education (SIWF),
2. Homepage of the Academy of Physicians (Austria) and
3. Homepage of the German Federal Medical Association (BAEK).

Further links were considered and the results were presented in tabular form. The assessment methods used in the specialist assessments are critically examined with regard to established quality criteria and recommendations for the further development of the specialist assessments are derived from these.

**Results:** The following assessment methods are already used in Switzerland and Austria: written examinations with multiple choice and short answer questions, structured oral examinations, the Script Concordance Test (SCT) and the Objective Structured Clinical Examination (OSCE). In some cases, these assessment methods are combined (triangulation). In Germany, on the other hand, the oral examination has so far been conducted in an unstructured manner in the form of a ‘collegial content discussion’. In order to test knowledge, practical and communicative competences equally, it is recommended to implement a triangulation of methods and follow the further recommendations described in this article.

**Conclusion:** While there are already accepted approaches for quality-assured and competence-based specialist assessments in Switzerland and Austria at present, there is still a long way to go in Germany. Following the recommendations presented in this article, a contribution could be made to improving the specialist assessments in the DACH region according to the specialist assessments objectives.

## Zusammenfassung

**Einleitung: **Facharztprüfungen in der Medizin haben die Aufgabe, sicherzustellen, dass Ärzte über die klinischen Kompetenzen verfügen, um ihr Fach eigenverantwortlich zu vertreten und Patienten eigenständig und bestmöglich nach dem aktuellen Stand des Wissens zu versorgen. Bisher gibt es zum Stand der Facharztprüfungen im deutschsprachigen Raum (DACH) keine umfassenden Darstellungen. Deshalb werden in diesem Artikel die zum Einsatz kommenden Prüfungsmethoden in der DACH-Region zusammengestellt, kritisch bewertet und Empfehlungen zur Weiterentwicklung beschrieben.

**Methoden: **Die Internetseiten der folgenden Institutionen wurden nach Informationen in Bezug auf die eingesetzten Prüfungsmethoden und die Organisation der Facharztprüfungen durchsucht:

1. Homepage des Schweizerischen Instituts für ärztliche Weiter- und Fortbildung (SIWF),
2. Homepage der Akademie der Ärzte (Österreich),
3. Homepage der deutschen Bundesärztekammer (BAEK).

Weiterführende Links wurden berücksichtigt und die erhaltenen Ergebnisse tabellarisch dargestellt. Die in den Facharztprüfungen eingesetzten Prüfungsmethoden werden hinsichtlich etablierter Qualitätskriterien kritisch betrachtet und daraus Empfehlungen zur Weiterentwicklung der Facharztprüfungen abgeleitet.

**Ergebnisse:** In der Schweiz und in Österreich kommen bereits folgende Prüfungsmethoden zum Einsatz: schriftliche Prüfungen mit Multiple Choice und Kurzantwort-Fragen, strukturierte mündliche Prüfungen, der Script Concordance Test (SCT) und die Objective Structured Clinical Examination (OSCE). Teilweise werden diese Prüfungsmethoden miteinander kombiniert (Triangulation). In Deutschland wird dagegen bisher unstrukturiert mündlich in Form eines "kollegialen Fachgesprächs" geprüft. Damit Wissen, praktische und kommunikative Kompetenzen gleichermaßen überprüft werden können, werden die Methoden-Triangulation und die Beachtung der in diesem Beitrag beschriebenen weiteren Empfehlungen nahegelegt.

**Schlussfolgerung: **Während es in der Schweiz sowie in Österreich bereits gute Ansätze für qualitätsgesicherte und kompetenzbasierte Facharztprüfungen gibt, ist man in Deutschland noch davon entfernt. Mit den in diesem Artikel vorgestellten Empfehlungen, könnte ein Beitrag dazu geleistet werden, die Facharztprüfungen in der DACH-Region ihren Zielen gemäß zu verbessern.

## Introduction

Assessments fulfil a wide range of important tasks: they have a strong effect on learning, they can be used to provide feedback on the effectiveness of education and training programmes and, consequently, they can help protect patients [1]. Until the middle of the twentieth century, medical assessments were mainly written in the form of essays or oral assessments [1], [2]. At that time, evaluations derived from assessments often turned out to be subjective, arbitrary and non-reproducible [1]. Subsequently, standardised tests such as multiple-choice examinations (MC examinations) or Objective Structured Clinical Examinations (OSCE) [3] were developed (Case & Swanson 1996, Norcini & Burch 2007, Kogan et al. 2009, cited after Norcini [1]). Tests should be objective, reproducible (reliable) and valid. Furthermore, they should be accepted by test takers and examiners, have a learning-promoting component and be as cost-efficient as possible [4]. “Objectivity” means that the test should be as independent as possible from the examiner as a person – their attitudes, feelings and motives. It refers to the performance, evaluation and interpretation of a test [5]. A test should provide approximately the same result when repeated; in other words it should be “reliable”. Reliability is a measure of the trustworthiness of a test. Reliability is represented as a coefficient ranging from 0 (no reliability) to 1 (perfect reliability). The value 0.80 is often set as the minimum standard for a significant high stakes test [6]. Among other things, a test is “valid” if it measures what it claims to measure. It is thus a measure of the measurement accuracy of a test [5]. It would be desirable for all valid tests to be compared to an external standard, but one is often not available in practice. In this case, expert assessments are often used for validation. In medical education, constructs are mainly used – in other words, abstract concepts and principles derived from behaviour and explained by pedagogical and psychological theories [7]. This fact is represented by the concept of “construct validity”. 

Society relies on tests that ensure that patients can place themselves in the care of competent and qualified physicians who have reached a minimum standard [1]. According to Premi, specialist examinations should ensure that colleagues who have passed this assessment have acquired the knowledge and necessary skills of their specialist group and can apply them independently (Premi 1994, quoted from Ratnapalan & Hilliard [8]. Examinations that demonstrate the necessary knowledge, skills and attitudes for the pursuit of a profession are part of self-regulation in continuing medical education. This is viewed with increasing scepticism worldwide, especially against the background that training to become a physician is a very expensive affair and is often financed by the public purse [9]. For this reason, governments in Australia, Great Britain and Canada are directly entrusted with regulating continuing medical education (Chantler & Ashton 2009, Shaw et al. 2009, Medicare Advisory Commission 2009, cited after Holmboe [9]). 

The official training regulations for postgraduate medical doctors, which were developed by the BAEK, have the character of a recommendation. Completion of the specialist training is assessed on the basis of documented competences, issued by the respective physician in charge, and an oral examination. According to the BAEK, this “certificate of further training” is proof of the acquired competence and serves as quality assurance for patient care and citizen orientation. The term ‘competence’ is not specified here [10]. 

In Switzerland, the FMH (Foederatio Medicorum Helveticorum) is the professional association of Swiss physicians. It is the umbrella organisation of more than 70 doctors' organisations. The Swiss Institute for Medical Continuing Education (SIWF) is an autonomous body of the FMH and ensures high-quality continuing education for doctors in over 120 specialist areas. In cooperation with the professional associations, the SIWF issues a detailed further training programme [https://www.siwf.ch/] for each specialist area. 

The Austrian Medical Association (ÖÄK) grants the right to practise as employed, self-employed and self-reliant physicians. The ÖÄK has entrusted the ‘Austrian Academy for Physicians GmbH [https://www.aerztekammer.at/] with the implementation of the medical examination as a prerequisite for the pursuit of a medical profession. The training contents and the corresponding certificates for the acquisition of a specialist title have been drawn up and specified by the ÖÄK [https://www.aerztekammer.at/ausbildungsinhalte-und-rasterzeugnisse-kef-und-rz-v-2015].

Over the last few years, competence orientation has increasingly come to the fore in medical education and postgraduate medical education. This is with the aim of ensuring that graduates master the challenges of practical work and possess all the necessary skills [11]. The physician competency framework “Canadian Medical Education Directives for Specialists” (CanMEDS) was developed by Frank et al. to guarantee comprehensive postgraduate training for physician [12]. On the basis of a systematic literature analysis and broad-based expert and stakeholder surveys, seven medical roles were defined by Frank et al. [11] to establish CanMEDS and integrate it into all of Canada’s continuing education programmes [13]:

1. Medical Expert
2. Communicator
3. Collaborator
4. Leader
5. Health Advocate
6. Scholar
7. Professional

Numerous key competencies are assigned to these roles. The CanMEDS role model has already been integrated into national learning objective catalogues for medical studies in Europe (Netherlands (Laan 2010, quoted from Jilg) [13], Switzerland [14], Germany [http://www.nklm.de]). Not only should medical teaching be competence-oriented, but also any successive further education. This includes a competence-oriented examination of knowledge, skills and attitudes.

To date, there exists no compilation of the extent to which the specialist assessments of the DACH region are competence-based and whether the quality criteria previously mentioned, such as objectivity and reliability, are taken into account. The aim of this work is therefore to provide an overview of the existing summative specialist assessment formats in the DACH region and their organisation, to take a critical look at the formats with regard to quality criteria and to make recommendations on the basis of the international literature. As a first step, this compilation should make the current situation better known and highlight possible directions for the further development of specialist assessments in the DACH region.

## Methods

The following homepages were searched for references to existing assessment formats and the organisation of specialist assessments:

1. Homepage of the Swiss Institute for Medical Education and Training [https://www.siwf.ch/]
2. Homepage of the Austrian Academy of Physicians [https://www.aerztekammer.at/]
3. Homepage of the German Federal Medical Association [10]

Using the websites of these national umbrella organisations, the contents of the homepages of professional associations or regional chambers of physicians were evaluated. These provided further information on the examination formats currently used. The quality of the Internet research therefore depends on the information and data listed there. A review of the individual annual reports of the State Medical Associations provided, as far as available, an overview of the number of examinations carried out in one year and the corresponding failure rates. Further key statistical figures or costs were not provided. 

Furthermore, the test methods used were critically evaluated with regard to the quality criteria of the tests (validity, reliability, objectivity, acceptance, cost efficiency and influence on learning). In addition, criteria for best practice specialist assessments were derived from the literature.

## Results

### Overview of the assessment formats used in the DACH region

#### Switzerland

In Switzerland, Multiple Choice (MC) examinations are used in 26 of 46 subject areas. The number of questions varies between 50 and 200. The minimum exam duration is 120 minutes; the maximum is 360 minutes. The specialist areas of Anaesthesiology, Allergology/Immunology, Cardiology and Vascular Surgery are examined in conjunction with the European Union of Medical Specialists (UEMS). The following types of questions are used in Switzerland, although there is a clear variance in their occurrence within the specialist areas: type A positive, type A negative, type Kprim, type B, type E, type R, and type Pick N are used as MC formats. Short Answer Questions (SAQ) and Script Concordance Tests (SCT) are also used. Swanson and Case [15] provide a good introduction to this, including examples for the different question types. In the field of Psychiatry/Psychotherapy, candidates must submit a written paper in part 2 and take part in a colloquium. In Radiology, for example, open text formats are used, but they are not explained in detail. Here the diagnosis of cases is in the foreground and a web-based examination tool is used. In addition to the written format, there are also oral assessments in Switzerland, including discussion of a paper, presentation of a patient case, holding a colloquium and structured oral assessments (SMP). The duration of these varies from 20 minutes to 180 minutes. Some subjects, e.g. Endocrinology/Diabetology, combine a written examination with an oral examination. In 23 subject areas, i.e. 50% of the specialist assessments in Switzerland, assessments with a practical component take place. In the fields of Oto-Rhino-Laryngology and Thoracic Surgery, for example, practical examinations are held as part of an operation. Rheumatology carries out an OSCE comprising 9 stations, with 10 minutes available per station. It is not only knowledge (Anatomy, Pathophysiology etc.), but also practical skills (examination techniques), as well as communicative skills, that are tested in a standardised way. In 2017, the SIWF awarded 1428 medical specialist titles [16] and, in 2018, 1434 medical specialist titles [17]. The SIWF does not publish a failure rate in its annual report.

##### Austria

14 of 57 tests are performed with MC questions. The minimum number of questions is 50 and they can reach up to 200 questions, which have to be answered in the field of skin and venereal diseases. The candidates have 60 to 300 minutes at their disposal. Anaesthesiology and Urology are affiliated to the European specialist examination. The Pathology and Radiology departments use tests with short answer questions, which last from 80 to 240 minutes. In Austria, 45 subject areas are examined orally on the basis of structured oral examinations (the use of a so-called “blueprint”: pre-formulated questions and a horizon of expectation). In this context, the term “blueprint” refers to a weighted assessment plan in which the selection of relevant examination content ensures that each candidate is treated equally in terms of that content. For most subjects, a blueprint is created and explicitly mentioned in the exam description. The duration of the examination can vary between 40 and 120 minutes. Some subjects are examined both in writing and orally. There are currently no clinical-practical examinations in Austria. We do not have statistics on the number of tests carried out in Austria per year and the corresponding failure rates.

##### Germany

The specialist assessment is held at all regional medical associations in the form of an unstructured oral examination (UMP), which lasts at least 30 minutes and can last up to 60 minutes. This type of examination is used for all medical specialist qualifications and is also referred to as a “collegial expert discussion”. The number of examiners may vary and at least one examiner must be from the field to be examined. The examination results must be documented. Typically, a structured blueprint is not prepared and the questions are not pre-formulated in advance (in the sense of a standardised and structured examination and a specified expectation horizon). The Landesärztekammer Hamburg is an exception: here, the questions are handed over in advance to the chairman of the examination. Table 1 (Tab. 1) shows the number of specialist examinations and the associated failure rates. The data were taken from the annual reports of the respective regional medical associations for the year 2017, which were available online. An inquiry to the BAEK regarding comprehensive, nationwide statistics revealed that such statistics were not available. 

#### Critical appraisal of the tests used in the DACH region

##### MC exams

MC examinations are widely used in medicine as an assessment method because they can be cost-efficient and can offer high validity and reliability for testing knowledge (Norcini 1985, cited after Gerhard-Szep [18]. This presupposes, however, that a sufficient number (at least 40) of high-quality questions (in content and form) are used per test (Jünger 2014, cited after Gerhard-Szep [18]). Case et al. emphasise that two criteria are necessary to develop a good question: the question must both examine relevant content and be well structured [15]. The development of MC questions at a qualitatively high level is time-consuming. With written examination methods it is above all possible to test factual knowledge. In contrast to an OSCE, it is not possible to test communicative and practical skills, or competences, using MC questions [19].

##### Short answer questions

For short answer questions, freely formulated, short, keyword-like answers must be given. Test takers must spontaneously think of the correct solution and cannot react to given answers [5]. This reduces the so-called “cueing” that gives candidates the opportunity to answer a question correctly without knowledge (Schuwirth 2004, cited from Epstein [20]). Ideally, “context-rich” question strains (case vignettes) are also offered here, which make it possible to test application knowledge and, for example, “clinical reasoning”. Reliability also depends to a large extent on the quality of evaluations carried out by the examiners [20] – in this case, training the examiners in advance can help. The evaluation is more susceptible to subjective distortions than with MC questions. Pre-formulated expectation horizons, to which the evaluators must orient themselves, can increase objectivity and should be available. Acceptable reliability values can be achieved by using several testers, each of whom is responsible for evaluating different tasks [5]. Rademakers et al. [21] provide a clear presentation of a task. In the meantime, there is also the possibility to evaluate computer-based answer options [22]. In the near future, new developments are to be expected in this area, which will make use of artificial intelligence methods.

##### Script Concordance Test (SCT)

**Figure 1:**
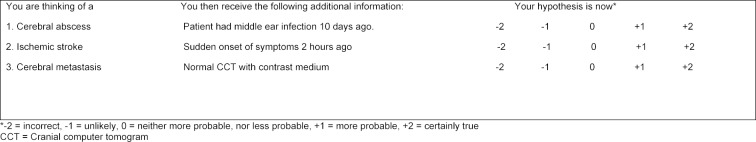
Case 1: A 75-year-old man presents with a right hemiparesis in the emergency room.

An SCT is used to check the ‘clinical reasoning’ competence of examinees in situations of clinical uncertainty [23]. Short clinical scenarios are described and additional information is provided step by step. In light of this new information, the investigator should then make diagnostic, follow-up or therapeutic decisions [24]. Using a 5-point Likert scale from -2 to +2, the examinee must indicate to what extent the additional information supports or does not support the disease hypothesis described in the scenario [25]. The results of the test takers are subsequently compared with the assessments of an expert group; the “gold standard” answer achieves the greatest number of points on which most experts have agreed [23]. Figure 1 (Fig. 1) shows an example of three questions [26].

Various working groups have been able to demonstrate the favourable psychometric properties of the SCT (construct validity, reliability and feasibility) [24]. Brailovsky et al. (2001, quoted after Epstein [20]) were able to show that the answers to such questions correlate with the candidate’s level of education and can predict their future performance in oral examinations in terms of their “clinical reasoning” ability [20]. A critical weakness of the 5-point Likert scale is that it can lead to misunderstandings and false assessments by the expert panel, so Lineberry et al. recommend the use of a 3-point scale consisting of the following: “refuted”, “neither refuted nor supported” and “applicable”. In addition, there is a risk that candidates’ answers will tend towards the middle and thus they will obtain a better test result than those who use the Likert scale in its extremes [26]. In addition, the usefulness of scores corresponding to an expert group is still under discussion, especially since 10-20 members [27] are recommended for this. The SCT is therefore quite complex.

##### Structured Oral Examination

The oral examination is a traditional form of examination in which one or more examiners address questions to the candidate. The oral exam is designed to evaluate knowledge, explore depth of knowledge and test other qualities such as mental agility. Colton & Peterson, Foster et al. and Kelly et al. criticised the use of oral examinations in high-stakes tests because of their low reliability (Peterson 1967, Foster et al. 1969, Kelly et al. 1971, cited after Davis [28]). During an oral examination numerous sources of error occur to which examiners are subject in the framework. For example, with the primacy effect, first impressions dominate over later impressions and, with the recency effect, later impressions are more lasting. In the halo effect, the perception and evaluation of one property outshines the perception and evaluation of other properties. Antipathy, sympathy and the composition of the examiners also have an influence on the evaluation of the test performance [29]. According to Roloff et al. reliability and objectivity increase when several examiners examine independently and the number of questions and the examination time increase (Roloff 2016, cited by Gerhard-Szep [18]). Memon et al. have named 15 quality assurance measures that are necessary from the point of view of the literature to ensure the objectivity, reliability and validity of specialist examinations. They state that the oral examination is most suitable for clinical reasoning and decision making. The content of the examination should be determined in advance by a panel of experts. The examination questions should be selected in such a way that they adequately examine not only the corresponding depth of knowledge but also the breadth of the subject area and guarantee a corresponding inter-item reliability. Examiners must first be trained with regard to carrying out an oral examination. Deviations between examiners (inter-examiner variations) must be monitored and addressed. Item creation and implementation processes must be standardised and a statistical evaluation should give conclusions about reliability. In the case of oral examinations, bias must be expected in the assessment and therefore quality assurance should be carried out to this end [30].

##### Unstructured Oral Examination

An unstructured oral examination is usually carried out by two untrained examiners who examine based on their experience. Typically, there is neither a pre-formulated expectation horizon nor previously written questions based on the curriculum or blueprint. As early as 1985, Jayawickramarajah et al. were able to demonstrate that two thirds of the questions in an unstructured oral examination exclusively examined factual knowledge. An additional problem of an unstructured oral examination is the high probability of an occurrence of Construct Irrelevant Variance (CIV) due to the fact that too few examiners are used. CIV could occur, for example, when the testing of the competence “clinical decision making” is influenced by the appearance, fear, language skills or clothing of the examinee. Construct Underrepresentation (CU) is a further hurdle that must be considered in the context of an unstructured oral examination, since, for example, two to three clinical scenarios that are tested cannot cover the entire range of the substance area to be tested. Concerns about the validity of this traditional form of examination have led to it being replaced by written examinations or structured oral examinations (Jayawickramarajah et al. 1985, Turnball, Danoff, & Norman, 1996, Pokorny & Frazier, 1966, quoted from Lamping et al. 2007 [31]).

##### OSCE

The OSCE test format was developed by Harden in the 1970s and primarily tests clinical and practical competencies. A higher objectivity is achieved through standardisation [3]. In order to achieve a standardised presentation of illnesses, actors are specially trained for this purpose [32]. A number of problems are presented to the examinee in the form of a course. Both the number of stations and the number of examiners have a positive effect on its reliability. Despite high variance in studies, a good reliability can already be achieved with more than 10 stations [33]. The examinee has approximately 5-15 minutes per station to complete the task [2]. The investigator checks the observed clinical competence using a checklist and/or a global rating scale. OSCEs allow a diagnosis to be made in the context of contact with the standardised patient (SP) through skilful anamnesis techniques and a patient-centred physical examination. According to Van der Vleuten and Tamblyn, trained SPs cannot be distinguished from real patients; they can repeatedly perform reliably, and they can also give valuable feedback to the test subjects (Van der Vleuten 1990, Tamblyn 1991, cited after Newble [2]). The OSCE is generally regarded positively by students and lecturers (Roberts & Brown 1990, quoted from Rushfort [34]), even if some students perceive the exam as stressful. Compared to other exams, it is more objective (Schuwirth & Van der Vleuten 2003, quoted after Rushforth [34]) and has a positive effect on the motivation of the candidates to study for it (Bartfey et al. 2004, quoted after Rushforth [34]).

#### Overview of the evaluation of the examination formats

Table 2 (Tab. 2), following Gerhard-Szep et al., Lubarsky et al., Epstein et al. and van der Vleuten et al. [4], [18], [20], [25] below, was designed to illustrate the most frequently occurring forms of assessment in the DACH region and to classify them from the authors’ point of view with regard to the essential quality criteria described. It essentially serves to provide a better overview and is intended to support the recommendations for a best practice specialist assessment.

#### Recommendation of a Best Practice Specialist Assessment

In the following, recommendations for a best practice specialist assessment are given. These have been derived from the current literature. 

Observance of the following recommendations helps to ensure that the resulting tests are as valid, reliable, objective, accepted, instructive and cost-effective as possible. This is necessary so that competence-oriented learning objectives can be meaningfully tested and examinations can prove that candidates have learnt the competences necessary for the independent treatment of patients.

1. **Use of different test methods (triangulation):** Different test methods should be used to adequately test knowledge, on the one hand, and practical and communicative skills on the other. Only the combination (triangulation) of the results of different assessment formats can ensure high validity and different competences [35].
2. **Prior definition of the contents and competences to be tested (blueprinting):** A weighted examination plan (so-called blueprint) provides a framework for the assessment by ensuring that a balanced selection of relevant learning objectives is incorporated into the test before it is held [36]. This is to ensure that the test is valid, fair, relevant and representative of the subject being examined.
3. **Prior definition of the questions and the horizon of expectations: **For oral and practical examinations, as for written formats, the questions and the horizon of expectations must be recorded in writing for each question/station in advance (so-called structuring). In oral and practical assessments, clearly structured checklists unequivocally present the horizon of expectations and thus ensure the necessary objectivity of interpretation and evaluation [18], [37].
4. **Sufficient number of questions, examiners, stations/learning objectives to be examined: **A minimum reliability of 0.8 is given for relevant assessments [4], [35]. In order to improve these, the number of tasks and/or their quality can be increased [35]. Likewise, the number of examiners has a positive effect on reliability (Swanson 1987, cited after Lynch) [38]. The more examiners test, the better the reliability becomes. In oral and practical examinations, it makes more sense to have one examiner per topic/station rather than several examiners at the same time with fewer stations/subjects.
5. **Quality assurance of the created questions/tasks: **The content and formal linguistic review and revision of the tasks is necessary to guarantee the unambiguity of the answers and the high quality of the tasks and questions. The validity of the examination results is strengthened by a review process, where experts trained in medical didactics review the questions and tasks [4], [39].
6. **Quality assurance in the evaluation of the assessment: **Quality assurance through test statistical evaluation of examinations makes it possible to revise OSCE stations and examination tasks in a targeted manner, to examine checklists and, if necessary, to draw conclusions about the quality of teaching. The following parameters are recommended: for written assessments at least one evaluation should be carried out with regard to reliability, selectivity and item difficulty (except for small numbers of candidates – i.e. less than 30 – because of the influence of chance). For oral or practical examinations, the better “OSCE-Metrics” [40] are very desirable for the evaluation of selectivity and item difficulty, as well as reliability at item level. A more modern approach to determining the pass mark for examinations, in which passing can also take place with more or less than 50% solved tasks, is that where the pass mark is determined in terms of content [36] – for example, a modified Angoff procedure with MC [41], or the borderline regression method with OSCE (see Wood et al. [42]).
7. **Learning effect for the candidates:** Assessments do not only serve for decision-making but they are also very important as a learning incentive for candidates and, additionally, they support the learning effect by giving the candidates feedback regarding their examination results [4]. For example, a feedback letter can be designed in such a way that the candidates know which areas of the blueprint they did less well in compared to the other tasks and the other candidates.

#### Consideration of cost efficiency

High quality assessments have their price, but they definitely represent a worthwhile investment with regard to the learning effect of test items [4], [39]. The method of examination should be chosen in each case, based on both its ability to adequately examine the subject matter (content validity) and it being as cost-efficient as possible. If, for example, it is primarily a question of testing the application knowledge of many candidates, a written examination with vignette questions is superior to a structured oral examination in terms of cost efficiency. The merger of professional societies can reduce the effort involved in, for example, practical examinations (like OSCE), with the aim of checking the CanMEDS roles (cf. the Swiss basic examination in surgery in the field of knowledge https://basisexamen.ch/).

## Discussion

In this paper, the question of which specialist assessment methods are used in the DACH region is examined. In addition, the assessment methods used are critically reviewed and recommendations for the further development of specialist assessments are described, based on the current literature.

### Testing methods used

More than 50% of the specialist assessments conducted in Switzerland take the form of MC examinations. The specialist areas of Anaesthesiology, Allergology/Immunology, Cardiology and Vascular Surgery are examined in conjunction with the European Union of Medical Specialists (UEMS). Other departments are planning to do so. Seven different types of MC questions are used, as well as the SAQ, free text examinations (not specified in more detail) and the SQT. Written work, SMP, practical examinations and an OSCE are also used. In total, 50% of the Swiss specialist examinations have a practical component (cf. attachment 1 and attachment 2 ).

In Austria, 25% of the specialist assessments are conducted as written examinations (MC questions). Two specialist areas examine in conjunction with the UEMS. SAQ and SMP are also related forms of examination. Blueprinting is used regularly. There is no practical examination yet (cf. attachment 2 and attachment 3 ). In Germany, an unstructured oral assessment takes place throughout the country, which is referred to as a “collegial content discussion“ (cf. attachment 4 ). 

#### Critical consideration and recommendations for the further development of specialist assessments

It is positive to note that in Switzerland 50% of the specialist assessments already have a practical component. In order to be able to test practical and communicative competences within the scope of the specialist assessment, a practical, communication and competence-oriented examination should be used in addition to a knowledge-oriented examination method (written/SMP). The OSCE format could be used here. In order to reduce costs, for example, at least parts of the examinations could be conducted nationwide. According to the literature, it is also positive to note that, in Switzerland and Austria, MC tests with type A pos. questions are used in the majority of cases to objectively test knowledge – including application knowledge – and evaluate it statistically. 

However, an assessment of the examined competence using these forms of examination is only possible if a detailed insight into the examinations is conducted and their results can be provided. The preparation of written examinations is often underestimated, and it is time-consuming, as review processes must take place in terms of content, formal language and (medical) didactics in order to guarantee the unambiguity of the answers provided. The workload is mainly shifted to the preparation phase (cf. Gerhard-Szep) [18]. In addition, ethical and cultural questions are often avoided when questions are created, since context-rich questions are difficult to write (Frederiksen 1984, cited from Epstein) [20]. Swing et al. generally recommend the use of regular examiner training as well as the use of expert groups that regularly critically question the examination method used [43]. Application knowledge does not have to be examined in writing, but it can also be examined in a structured, oral way. In addition to application knowledge, structured oral assessments can evaluate clinical decision making, professional thinking and self-confidence [30]. It should be borne in mind that every structured oral assessment is associated with high costs due to the high space and personnel requirements. Blueprinting and the creation of an expectation horizon are also necessary. Duration, number and experience of the examiners have a direct influence on the quality criteria of the structured oral assessment (Roloff 2016, cited after Gerhard-Szep [18]). Examiner training can help to raise awareness and reduce the psychological sources of error (see Kugler 2007 [29]) to which examiners are unconsciously subject. Here, resistance must be expected from previous examiners, who have tested for years without having been trained to do so.

In Germany, oral examinations are conducted in an unstructured manner, although the German Medical Association expressly emphasises on its website that the continuing education designation serves as proof of the acquired competence and quality assurance of patient care and citizen orientation [44]. The current specialist examinations in Germany therefore do not meet this requirement, as unstructured oral assessments do not fulfil the required quality criteria (also see table 2 (Tab. 2)). It can therefore only be assumed that the UMP has been developed following a tradition and has not yet been subject to a critical review. Therefore, the unstructured oral assessments cannot be recommended. In order to better fulfil their responsibility for medical quality assurance in the future, a first step could be to establish contact with medical faculties that have been gathering experience in the implementation of structured examination methods for several years. An exchange of experience could also take place with experts from the DACH region who are already carrying out quality assurance for the specialist assessments used. In order to convert unstructured oral examinations into structured oral examinations at short notice, the regional medical associations in Germany could get in touch with colleagues trained in medical didactics to conduct examiner training on site. Flum also emphasises the aspect of quality assurance by saying that it would be helpful to standardise competences and testing methods in postgraduate medical training in general medicine within the EU in order to ensure the quality of treatment and patient safety (45). Likewise, specialist assessments should be seen as an instrument for regulating the content of continuing education and should meet the actual care needs of the population as well as the learning objectives and curriculum [45].

A recommendation with regard to a best practice specialist assessment is made against the background that, so far, only a few articles have taken a stand on assessment methods in the field of postgraduate medical education. A combined use of assessment methods (triangulation) is indispensable in order to be able to cover the necessary competence spectrum. Likewise, the following should be used and documented: obligatory blueprinting (Dauphinee 1994, cited from Wass), the preparation of questions and horizon of expectations in advance, a sufficient number of questions/tasks, quality assurance measures relating to the preparation and evaluation of examination questions/tasks, examination feedback (Gronlund 1998, cited from Norcini) and the most cost-effective assessment methods possible. These recommendations are supported by numerous publications [1], [37], [46]. Caraccio et al. emphasise that different assessment methods should be combined so that the competence level of continuing training assistants can be assessed (Caraccio 2013, quoted from Flum [45]. Taylor et al. point to a necessary standardisation of specialist assessments, the costs of which must, however, be considered (Taylor 1998, cited after Flum [46]). David et al. and Adler et al. rightly discuss at this point that costs incurred in the context of continuing training must be reflected in the Diagnosis Related Group (DRG) system [47], [48]. The necessary use of blueprinting in the field of postgraduate medical education is supported by Wass et al. In order to optimise learning success during postgraduate medical education, competences should be continuously recorded using various methods and feedback given through formative assessments (e.g. Mini Clinical Examination (Mini-CEX), Direct Observation of Procedural Skills (DOPS), Portfolios etc.) [1], [49]. Competency-based curricula should take into account how knowledge, skills and attitudes are tested at the highest examination level: “does”, according to Miller (Miller 1990 [50]). Examinations should be competence-based [11]. Currently, so-called EPAs (Entrustable Professional Activities) are increasingly being used to support curriculum development and test new ways of competence-based learning and testing [51]. Such a professional activity (EPA) could be, for example, the identification of an emergency patient on a normal ward and the initial assessment and initiation of necessary medical measures. A special feature of the EPA approach is the assessment of the learner on the basis of the presumed need for supervision (“entrustment”). The use of EPAs is often intuitively attractive for clinically active physicians, but their potential, including existing challenges (see also literature on workplace-based assessments [51], must be further investigated before the replacement of quality-assured summative specialist examinations could be considered. 

On the one hand, this work is certainly limited by the fact that the data researched on the Internet can only be presented descriptively. On the other hand, continuing education curricula, the implementation of continuing education and the design of examinations are closely interlinked in the sense of “constructive alignment”. A further limitation is therefore that, in this article, we have primarily dealt with the presentation of the examinations used in the DACH region and that curricula on further specialist training could only be mentioned in passing. However, a follow-up article could explain the different curricula on further specialist training and their implementation within the DACH region. 

## Conclusions

In the DACH region, the organisation of specialist assessments, the assessment methods used and the quality assurance measures are very different. In contrast to Germany, structured and standardised specialist assessment methods are already used in Austria and Switzerland – as well as practical examinations in the latter. If specialist assessments are to ensure that specialist doctors have the necessary competences for patient care, they must also be designed in such a way that they can actually test competences. This currently appears not to be the case in all specialist areas in Germany, but also in most specialist areas in Austria and Switzerland. Therefore, in order to ensure the quality of postgraduate medical training, it is necessary that even more attention is paid in the three countries to the fact that summative specialist assessments also examine the intended competences of the prospective specialists. A combination of a written examination with a practical examination (e.g. OSCE) is currently recommended, as this not only tests knowledge but also other competences including practical and communicative skills (see table 2 (Tab. 2)).

## Acknowledgements

We thank Dr. med. Susanne Frankenhauser, MME, Dr. med. Uta Krämer and Brian Webber for their constructive help in reviewing the manuscript. 

## Competing interests

The authors declare that they have no competing interests. 

## Supplementary Material

Overview of oral assessments taking place in Switzerland

Overview of written examinations taking place in the DACH region

Overview of oral examinations taking place in Austria

Overview of oral assessments taking place in Germany

## Figures and Tables

**Table 1:**
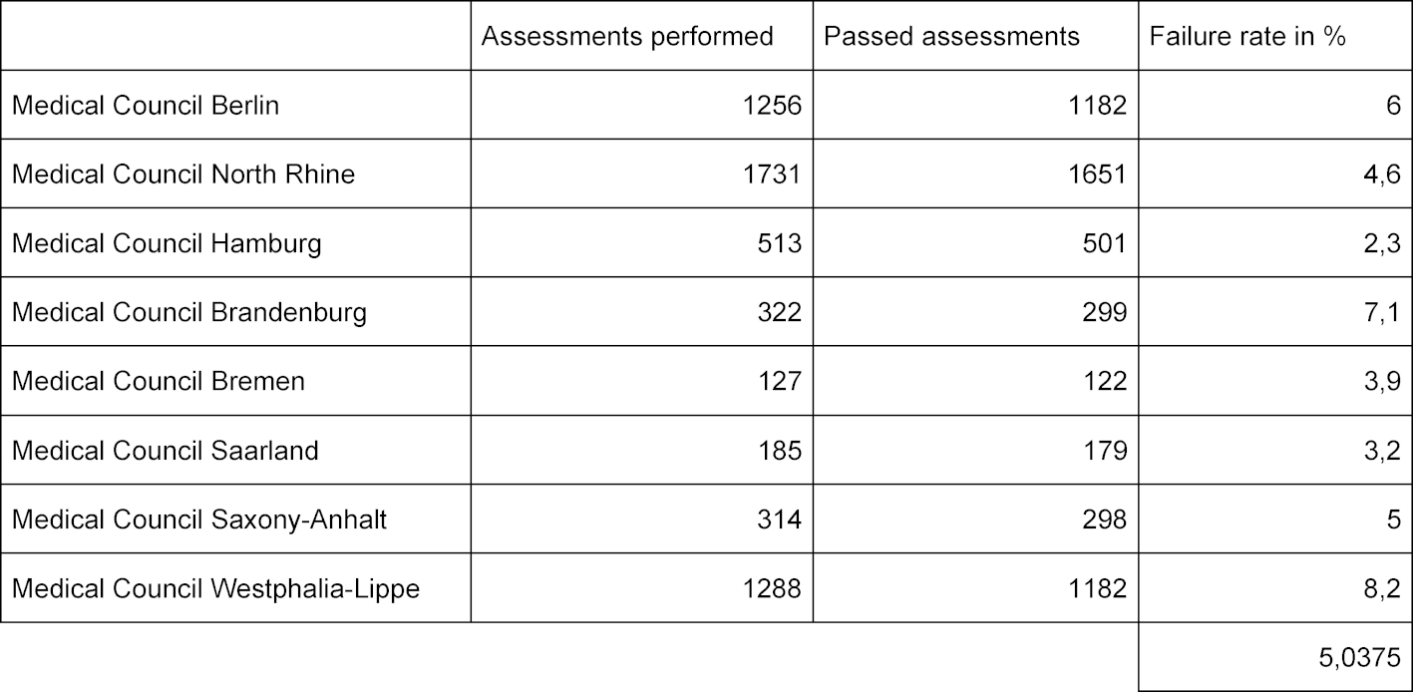
Overview of the key audit figures in the annual reports of the regional medical associations for the year 2017.

**Table 2:**
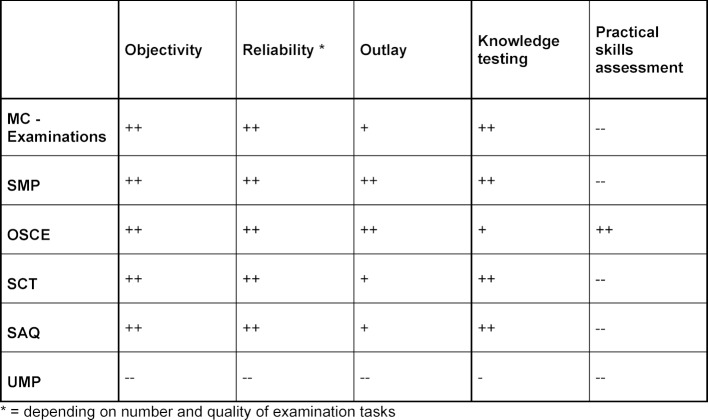
Presentation of the assessment methods used in the DACH region with regard to relevant characteristics, from “++” (=high) to “—” (=low) or suitable to unsuitable from the authors' point of view.

## References

[1] Norcini J, Anderson B, Bollela V, Burch V, Costa MJ, Duvivier R (2011). Criteria for good assessment: Consensus statement and recommendations from the Ottawa 2010 Conference. Med Teach.

[2] Newble D (2004). Techniques for measuring clinical competence: objective structured clinical examinations. Med Educ.

[3] Harden MR, Stevenson M, Downie WW, Wilson GM (1975). Assessment of clinical competence using objective structured examination. Br Med J.

[4] van der Vleuten CP (1996). The assessment of professional competence: Developments, research and practical implications. Adv Heal Sci Educ.

[5] Fabry G (2008). Medizindidaktik: ein Handbuch für die Praxis.

[6] van der Vleuten CP, Schuwirth LW (2005). Assessing professional competence: From methods to programmes. Med Educ.

[7] Downing S (2003). Validity: on the meaning ful interpretation of assessment data. Med Educ.

[8] Ratnapalan S, Hilliard R (2002). Needs Assessment in Postgraduate Medical Education: A Review. Med Educ Online.

[9] Holmboe ES, Sherbino J, Long DM, Swing SR, Frank JR (2010). The role of assessment in competency-based medical education. Med Teach.

[10] Bundesärztekammer (2018). (Muster-)Weiterbildungsordnung 2018.

[11] Frank J, Snell L, Sherbino J (2015). CanMEDS 2015 Physician Competency Framework.

[12] Kadmon M, Ganschow P, Gillen S, Hofmann HS, Braune N, Johannink J, Kühn P, Buhr HJ, Berberat PO (2013). Der kompetente Chirurg. Chirurg.

[13] Jilg S, Möltner A, Berberat P, Fischer MR, Breckwoldt J (2015). How do Supervising Clinicians of a University Hospital and Associated Teaching Hospitals Rate the Relevance of the Key Competencies within the CanMEDS Roles Framework in Respect to Teaching in Clinical Clerkships?. GMS Z Med Ausbild.

[14] Bürgi H, RindlisbacherB, Bader C, Bloch R, Bosman F, Gasser C, Gerke W, Humair JP, Im Hof V, Kaiser H, Lefebvre D, Schläppi P, Sottas B, Spinas GA, Stuck AE (2007). Swiss Catalogue of Learning Objectives for Undergraduate Medical Training.

[15] Case SM, Swanson DB (2002). Constructing Written Test Questions For the Basic and Clinical Sciences.

[16] Schweizer Institut für ärztliche Weiter- und Fortbildung (2017). Fortbildung: Investition in die Zukunft.

[17] Schweizer Institut für ärztliche Weiter- und Fortbildung (2018). Weiterbildungsgänge für weitere sieben Jahre akkreditiert.

[18] Gerhard-Szep S, Guentsch A, Pospiech P, Soehnel A, Scheutzel P, Wassmann T, Zahn T (2016). Assessment formats in dental medicine: An overview. GMS J Med Educ.

[19] Chenot JF, Ehrhardt M (2003). Objective structured clinical examination (OSCE) in der medizinischen Ausbildung: Eine Alternative zur Klausur. Z Allgemeinmed.

[20] Epstein RM (2007). Medical education - Assessment in medical education. N Engl J Med.

[21] Rademakers J, Ten Cate TJ, Bär PR (2005). Progress testing with short answer questions. Med Teach.

[22] Smith S, Kogan JR, Berman NB, Dell MS, Brock DM, Robins LS (2016). The development and preliminary validation of a rubric to assess medical students' written summary statements in virtual patient cases. Acad Med.

[23] Lubarsky S, Dory V, Duggan P, Gagnon R, Charlin B (2013). Script concordance testing: From theory to practice: AMEE Guide No. 75. Med Teach.

[24] Charlin B, Roy L, Brailovsky C, Goulet F, van der Vleuten C (2000). The Script Concordance Test: A Tool to Assess the Reflective Clinician. Teach Learn Med.

[25] Lubarsky S, Charlin B, Cook DA, Chalk C, van der Vleuten CP (2011). Script concordance testing: A review of published validity evidence. Med Educ.

[26] Lineberry M, Kreiter CD, Bordage G (2013). Threats to validity in the use and interpretation of script concordance test scores. Med Educ.

[27] Dory V, Gagnon R, Vanpee D, Charlin B (2012). How to construct and implement script concordance tests: Insights from a systematic review. Med Educ.

[28] Davis MH, Karunathilake I (2005). The place of the oral examination in today's assessment systems. Med Teach.

[29] Kugler, Schutz A (2007). Mündliche Prüfung Bankfachwirt. Mündliche Prüfung Bankfachwirt.

[30] Memon MA, Joughin GR, Memon B (2010). Oral assessment and postgraduate medical examinations: Establishing conditions for validity, reliability and fairness. Adv Heal Sci Educ.

[31] Lamping DL (2007). Assessment in health psychology. Can Psychol.

[32] Barrows HS (1993). An overview of the uses of standardized patients for teaching and evaluating clinical skills. Acad Med.

[33] Brannick MT, Erol-Korkmaz HT, Prewett M (2011). A systematic review of the reliability of objective structured clinical examination scores. Med Educ.

[34] Rushforth HE (2007). Objective structured clinical examination (OSCE): Review of literature and implications for nursing education. Nurse Educ Today.

[35] Möltner A, Schellberg D, Jünger J (2006). Grundlegende quantitative Analysen medizinischer Prüfungen. GMS Z Med Ausbild.

[36] Hays R (2008). Assessment in medical education: roles for clinical teachers. Clin Teach.

[37] JüngerJ, Just I (2014). Empfehlungender Gesellschaft für Medizinische Ausbildungund des Medizinischen Fakultätentagsfür fakultätsinterne Leistungsnachweise während des Studiums der Human-, Zahn- und Tiermedizin. GMS Z Med Ausbild.

[38] Lynch DC, Surdyk PM, Eiser AR (2004). Assessing professionalism: A review of the literature. Med Teach.

[39] van der Vleuten CP, Verwijnen GM (1996). Fifteen years of experience with progress testing in a problem-based learning curriculum. Med Teach.

[40] Pell G, Fuller R, Homer M, Roberts T, International Association for Medical Education (2010). How to measure the quality of OSCE: a review of metrics. Med Teach.

[41] Norcini JJ (2003). Standard setting on educational tests. Med Educ.

[42] Wood TJ, Humphrey-Murto SM, Norman GR (2006). Standard setting in a small scale OSCE: A comparison of the modified borderline-group method and the borderline regression method. Adv Heal Sci Educ Theory Pract.

[43] Swing SR, Clyman SG, Holmboe ES, Williams RG (2009). Advancing Resident Assessment in Graduate Medical Education. J Grad Med Educ.

[44] Bundesärztekammer (2015). Ärztliche Ausbildung in Deutschland. Weiterbildung.

[45] Flum E, Maagaard R, Godycki-Cwirko M, Scarborough N, Scherpbier N, Ledig T, Roos M, Steinhäuser J (2015). Assessing family medicine trainees--what can we learn from the European neighbours?. GMS Z Med Ausbild.

[46] Wass V, van der Vleuten C, Shatzer J, Jones R (2001). Assessment of clinical competence. Lancet.

[47] Adler G, von dem Knesebeck J, Hänle MM (2008). Qualität der medizinischen Aus-, Fort- und Weiterbildung. Z Evid Fortbild Qual Gesundhwes.

[48] David DM, Euteneier A, Fischer MR, Hahn EG, Johannink J, Kulike K, Lauch R, Lindhorst E, Noll-Hussong M, Pinilla S, Weih M, Wennekes V (2013). Die Zukunft der ärztlichen Weiterbildung in Deutschland - Positionspapier des Ausschusses Weiterbildung der Gesellschaft für medizinische Ausbildung (GMA). GMS Z Med Ausbild.

[49] Driessen E, Scheele F (2013). What is wrong with assessment in postgraduate training? Lessons from clinical practice and educational research. Med Teach.

[50] Mulder H, Ten Cate O, Daalder R, Berkvens J (2010). Building a competency-based workplace curriculum around entrustable professional activities: The case of physician assistant training. Med Teach.

[51] O'Dowd E, Lydon S, O'Connor P, Madden C, Byrne D (2019). A systematic review of 7 years of research on entrustable professional activities in graduate medical education, 2011-2018. Med Educ.

