# Supplementary material for: Assessment methods in medical specialist assessments in the DACH region – overview, critical examination and recommendations for further development
Source: GMS J Med Educ. 2019 Nov 15;36(6):Doc78. doi: 10.3205/zma001286 (PMC6905366; doi:10.3205/zma001286)
Supplement: Overview of oral assessments taking place in Switzerland [file JME-36-6-78-s-001.pdf]

Description	Duration*	Specialist field	Country	Source
Case based discussion, (Swiss Catalogue of Objectives in Anaesthesiology and Resuscitation SCOAR)	2 x 30	Anaesthesiology		http://www.sgar-ssar.ch/weiterbildung/fachexamen/
		Angiology	CH	http://www.fmh.ch/bildung-siwf/fachgebiete/facharzt-titel-und-schwerpunkte/angiologie.html
		Allergology / Clinical Immunology		http://www.ssai.ch/index.php?id=22&L=2%252Fembed%252Fday
6 standardised patients - case studies	3 x 30	Surgery	CH	www.Basisexamen.ch
Oral assessments	180	Endocrinology / Diabetology	CH	http://www.fmh.ch/bildung-siwf/fachgebiete/facharzttitel-und-schwerpunkte/endokrinologie-diabetologie.html
Case discussion		Gastroenterology	CH	http://www.fmh.ch/bildung-siwf/fachgebiete/facharzttitel-und-schwerpunkte/gastroenterologie.html
		Gynaecology / Obstetrics	CH	http://www.fmh.ch/bildung-siwf/fachgebiete/facharzttitel-und-schwerpunkte/gastroenterologie.html
SMP	20–40		CH	http://www.sgh-ssh.ch/weiterfortbildung/weiterbildung/facharztexamen
	3x30	Haematology	CH	https://www.ebcts.org/examination/
	30-60	Cardiac and Thoracic Vascular surgery	CH	http://www.fmh.ch/files/pdf14/infektiologie_version_internet_d.pdf
Guided Questions	4 x 25	Infectiology	CH	https://www.esahq.org/~/media/ESA/Files/EDUCATION/EDAIC%20Part%20II/Diploma%20Guide%20-%20German.ashx
Oral practical examination, 3 patients, case vignettes, video examples		Intensive Care	CH	http://www.swiss-paediatrics.org/sites/default/files/2016_informationen_facharztpruefung_d.pdf
	60	Paediatrics and Juvenile Medicine	CH	http://www.fmh.ch/files/pdf17/medizinische_genetik_version_internet_d.pdf
Case studies		Medical Genetics	CH	http://www.sgmo.ch/weiterbildung/facharztpruefung/
	40	Medical Oncology	CH	http://www.swissnephrology.ch/media/Continued_Education/FMH_Folder/Ablauf-Deroulement_DEFR_2016.pdf
		Nephrology	CH	http://www.swissneurosurgery.ch/exams
SMP, case vignettes	60	Neurosurgery	CH	http://www.swissneuro.ch/download/Content/facharztpruefung/SNG Prufungsreglement%2DFacharztprufungFMHNeurologie2012 Revision2011u.2012DFInternetneu.pdf
Discussion of a paper, questions on 2 core topics	30+15	Neurology	CH	http://www.fmh.ch/files/pdf18/pharmazeutische_medizin_version_internet_d.pdf
2 patient dossiers, evaluation according to defined horizon of expectations	2 x 25	Pharmaceutical Medicine	CH	http://www.reha-schweiz.ch/fileadmin/Dateien/Facharztweiterbildung/Facharztpruefungen/Durchfuehrungsbestimmungen_der_Pruefungs-kommission_SGPMR.pdf
	2 x 25	Physical Medicine & Rehabilitation	CH	http://www.ebopras.org/downloads/2016/2016_09_EBOPRAS_Examination_Rules_&_Info.pdf
Colloquium, tape recording	30	Plastic, Reconstructive / Aesthetic Surgery	CH	http://sgr-ssr.ch/facharztpruefung/
SMP		Psychiatry / Psychotherapy	CH	http://www.fmh.ch/files/pdf16/thoraxchirurgie_version_internet_d.pdf
Presentation of patient dossiers and case studies		Thoracic Surgery	CH	http://www.tropenmedizin-fmh.ch/sample%20questions.pdf

* = in minutes
