# Supplementary material for: Assessment methods in medical specialist assessments in the DACH region – overview, critical examination and recommendations for further development
Source: GMS J Med Educ. 2019 Nov 15;36(6):Doc78. doi: 10.3205/zma001286 (PMC6905366; doi:10.3205/zma001286)
Supplement: Overview of written examinations taking place in the DACH region [file JME-36-6-78-s-002.pdf]

Number of Questions*	Duration**	Description	Specialist field	Country	Source
120	120	Apos., EDAIC***	Anaesthesiology	CH	http://www.sgar-ssar.ch/weiterbildung/fachexamen/
100		EAACI***	Allergology / Clinical Immunology	CH	http://www.eaaci.org/activities/eaaci-exam/upcoming-exam.html
100		20 SCT	Internal Medicine	CH	http://www.fmh.ch/bildung-siwf/fachgebiete/facharzttitel-und-schwerpunkte/allgemeine-innere-medizin.html
			Angiology	CH	http://www.fmh.ch/bildung-siwf/fachgebiete/facharzttitel-und-schwerpunkte/angiologie.html
150	240		Surgery	CH	https://www.fmh.ch/siwf/siwf/weiterbildung/facharzttitel-und-schwerpunkte/chirurgie.cfm#i113313
	240	Written examination	Endocrinology / Diabetology	CH	http://www.fmh.ch/bildung-siwf/fachgebiete/facharzttitel-und-schwerpunkte/endokrinologie-diabetologie.html
200	360		Gastroenterology	CH	http://www.ebghe.eu/examformat/Default.aspx
		FEBVS***	Vascular Surgery	CH	http://www.uemsvascular.com/febvs.php
			Gynaecology / Obstetrics	CH	http://www.fmh.ch/bildung-siwf/fachgebiete/facharzttitel-und-schwerpunkte/gynaekologie-geburtshilfe.html
60	120		Hand Surgery	CH	Calcagni M. The European Board Of Hand Surgery Examination. J Hand Surg Eur. 2013;38(6):692-695
120	240		Intensive Care	CH	https://www.esahq.org/~/media/ESA/Files/EDUCATION/EDAIC%20Part%20II/Diploma%20Guide%20-%20-German.ashx
90	180	EEGC***	Cardiology	CH	http://www.swisscardio.ch/DOCS_PUBLIC/Fort_Weiterbildung/WB_Programm_D_2015.pdf http://www.fmh.ch/bildung-siwf/fachgebiete/facharzttitel-und-schwerpunkte/kardiologie.html http://www.uems-cardio.eu/jart/prj3/uems/main.jart?rel=de&content-id=1395393952438&reserve-mode=active
60		60 questions plus SAQ	Children/Youth Medicine	CH	http://www.swiss-paediatrics.org/sites/default/files/2016_informationen_facharztpruefung_d.pdf
120		Typ A, Typ Kprim, SAQ	Clinical Pharmacology / Toxicology	CH	http://www.clinpharm.ch/Pharmaco/FichierComite/SOP-specialist-exam-2013-V2.pdf
100	150	62 Typ A, 18 Typ B, 20 Typ Kprim	Medical Oncology	CH	http://www.esmo.org/Career-Development/ESMO-Examination
			Neurosurgery	CH	http://www.swissneurosurgery.ch/exams
100	240	Typ A und Kprim	Neurology	CH	http://www.swissneuro.ch/download/Content/facharztpruefung/SNGPrufungsreglement%2DFacharztprufung FMHNeurologie2012Revision2011u.2012DFInternetneu.pdf
		Typ A, Typ B, Typ E, Typ Kprim,	Nuclear medicine	CH	http://www.nuklearmedizin.ch/images/2015_08_18_Gegenstandskatalog_FMH_Radiologie_1.Teilpruefung.pdf

60 -100	240		Otho-Rhino-Laryngology	CH	https://www.orl-hno.ch/fileadmin/user_upload/Dokumente/Bildung/Weiterbildung/Pruefungen/Pruefungen_DE/ORL_FA_Weiterbildungsprogramm_DE.pdf
120	240		Pharmaceutical Medicine	CH	http://www.fmh.ch/files/pdf18/pharmazeutische_medizin_version_internet_d.pdf
100	180	Typ A, Typ A-, Typ B, Typ E, Typ Kprim, Typ R, Typ Pick N	Physical Medicine / Rehabilitation	CH	http://www.reha-schweiz.ch/fileadmin/Dateien/Facharztweiterbildung/Facharztpruefungen/Durchfuehrungs-bestimmungen_der_Pruefungskommission_SGPMR.pdf
120	180		Plastic, Reconstructive / Aesthetic Surgery	CH	http://www.ebopras.org/downloads/2016/2016_09_EBOPRAS_Examination_Rules_&_Info.pdf
100	240	Typ A pos., Typ A negativ, Typ E, Typ Kprim	Psychiatry / Psychotherapy	CH	http://www.psychiatrie.ch/sgpp/weiterbildung/facharztpruefungen/facharztpruefung-1-teil/index.php?eID=tx_securedownloads&u=0&g=0&t=1474887327&hash=9332dcfa73b50525daa1291d4853d65980ddff3a&file=/fileadmin/SGPP/user_upload/FAP/d_Informationen_FAP_I.pdf
			Radiology	CH	http://sgr-ssr.ch/facharztpruefung/
50	165	Incl. dossier and case vignettes	Tropical/Travel Medicine	CH	http://www.tropenmedizin-fmh.ch/sample%20questions.pdf
100	120		Urology	CH	http://www.ebu.com/examinations/
120	120	Typ A pos., EDAIC***	Anaesthesiology / Intensive Care Medicine	A	https://www.arztakademie.at/pruefungen/oeaek-facharztpruefung/informationen-zu-den-einzelnen-sonderfaechern/anaesthesiologie-und-intensivmedizin/
120	240	Typ A pos., Blueprint	Ophthalmology/ Optometry	A	https://www.arztakademie.at/pruefungen/oeaek-facharztpruefung/informationen-zu-den-einzelnen-sonderfaechern/augenheilkunde-und-optometrie/
100	180	Typ A pos., Blueprint	Surgery	A	https://www.arztakademie.at/pruefungen/oeaek-facharztpruefung/informationen-zu-den-einzelnen-sonderfaechern/chirurgie/
120	240	Typ A pos. Blueprint	Gynaecology / Obstetrics	A	https://www.arztakademie.at/pruefungen/oeaek-facharztpruefung/informationen-zu-den-einzelnen-sonderfaechern/frauenheilkunde-und-geburtshilfe/
200	240	Typ A pos.	Skin / Venereal Diseases	A	https://www.arztakademie.at/pruefungen/oeaek-facharztpruefung/informationen-zu-den-einzelnen-sonderfaechern/haut-und-geschlechtskrankheiten/
150	300	Typ A pos., Blueprint	Internal Medicine	A	https://www.arztakademie.at/pruefungen/oeaek-facharztpruefung/informationen-zu-den-einzelnen-sonderfaechern/innere-medizin/
120	240	Typ A pos., Typ A neg., Blueprint	Internal Medicine / Pneumology	A	https://www.arztakademie.at/pruefungen/oeaek-facharztpruefung/informationen-zu-den-einzelnen-sonderfaechern/innere-medizin-und-pneumologie/

120	240	Typ A pos., Blueprint	Childhood / Adolescent Diseases	A	https://www.arztakademie.at/pruefungen/oeaek-facharztpruefung/informationen-zu-den-einzelnen-sonderfaechern/kinder-und-jugendheilkunde/
120	240	Typ A pos., Typ A neg., Blueprint	Lung Diseases	A	https://www.arztakademie.at/pruefungen/oeaek-facharztpruefung/informationen-zu-den-einzelnen-sonderfaechern/lungenkrankheiten/
100	180	Typ A pos., Blueprint	Orthopaedics / Orthopaedic Surgery	A	https://www.arztakademie.at/pruefungen/oeaek-facharztpruefung/informationen-zu-den-einzelnen-sonderfaechern/orthopaedie-und-orthopaedische-chirurgie/
120	240	Typ A pos., Blueprint	Orthopaedics / Traumatology	A	https://www.arztakademie.at/pruefungen/oeaek-facharztpruefung/informationen-zu-den-einzelnen-sonderfaechern/orthopaedie-und-traumatologie/
50	60	Typ A pos., Kprim, Blueprint	Radiology	A	https://www.arztakademie.at/pruefungen/oeaek-facharztpruefung/informationen-zu-den-einzelnen-sonderfaechern/radiologie/
120	240	Typ A pos., 4 diagnostic imaging questions, blueprint	Trauma Surgery	A	https://www.arztakademie.at/pruefungen/oeaek-facharztpruefung/informationen-zu-den-einzelnen-sonderfaechern/unfallchirurgie/
100	120	EBU***	Urology	A	https://www.arztakademie.at/pruefungen/oeaek-facharztpruefung/informationen-zu-den-einzelnen-sonderfaechern/urologie/

* = MC questions ** = in minutes *** = Examination of the European Union of Medical Specialists
