# Supplementary material for: Assessment methods in medical specialist assessments in the DACH region – overview, critical examination and recommendations for further development
Source: GMS J Med Educ. 2019 Nov 15;36(6):Doc78. doi: 10.3205/zma001286 (PMC6905366; doi:10.3205/zma001286)
Supplement: Overview of oral examinations taking place in Austria [file JME-36-6-78-s-003.pdf]

Description	Duration*	Specialist field	Country	Source
SMP		General Surgery / Vascular Surgery	A	https://www.arztakademie.at/pruefungen/oeaek-facharztpruefung/oeaek-pruefungstermine-2017/
		Anatomy	A	
SMP, 4 case studies, rotation from one station to another (4 stations), Blueprint	4 x 10	Anaesthesiology / Intensive Care Medicine	A	https://www.arztakademie.at/pruefungen/oeaek-facharztpruefung/informationen-zu-den-einzelnen-sonderfaechern/anaesthesiologie-und-intensivmedizin/
SMP, Blueprint	60 - 90	Occupational medicine	A	https://www.arztakademie.at/pruefungen/oeaek-facharztpruefung/informationen-zu-den-einzelnen-sonderfaechern/arbeitsmedizin/
SMP, Blueprint		Blood Group Serology / Transfusion Medicine	A	https://www.arztakademie.at/pruefungen/oeaek-facharztpruefung/informationen-zu-den-einzelnen-sonderfaechern/blutgruppenserologie-und-transfusionsmedizin/
SMP, 6 Stations and groups of 6 candidates each		Surgery	A	https://www.arztakademie.at/pruefungen/oeaek-facharztpruefung/informationen-zu-den-einzelnen-sonderfaechern/chirurgie/
SMP		Forensic Medicine	A	https://www.arztakademie.at/pruefungen/oeaek-facharztpruefung/informationen-zu-den-einzelnen-sonderfaechern/gerichtsmedizin/
SMP, Blueprint	80	Ear, Nose and Throat Diseases	A	https://www.arztakademie.at/pruefungen/oeaek-facharztpruefung/informationen-zu-den-einzelnen-sonderfaechern/hals-nasen-und-ohrenheilkunde/
SMP, Blueprint		Cardiac Surgery	A	https://www.arztakademie.at/pruefungen/oeaek-facharztpruefung/informationen-zu-den-einzelnen-sonderfaechern/herzchirurgie/
SMP		Histology / Embryology	A	https://www.arztakademie.at/pruefungen/oeaek-facharztpruefung/informationen-zu-den-einzelnen-sonderfaechern/histologie-und-embryologie/
SMP, Blueprint		Hygiene / Microbiology	A	https://www.arztakademie.at/pruefungen/oeaek-facharztpruefung/informationen-zu-den-einzelnen-sonderfaechern/hygiene-und-mikrobiologie/
SMP, Blueprint	120	Immunology	A	https://www.arztakademie.at/pruefungen/oeaek-facharztpruefung/informationen-zu-den-einzelnen-sonderfaechern/immunologie/
SMP, Blueprint		Internal Medicine / Angiology	A	https://www.arztakademie.at/pruefungen/oeaek-facharztpruefung/informationen-zu-den-einzelnen-sonderfaechern/innere-medizin-und-angiologie/
SMP, Blueprint		Internal Medicine / Endocrinology/ Diabetology	A	https://www.arztakademie.at/pruefungen/oeaek-facharztpruefung/informationen-zu-den-einzelnen-sonderfaechern/innere-medizin-und-endokrinologie-und-diabetologie/
SMP, Blueprint		Internal Medicine / Gastroenterology / Hepatology	A	https://www.arztakademie.at/pruefungen/oeaek-facharztpruefung/informationen-zu-den-einzelnen-sonderfaechern/innere-medizin-und-gastroenterologie-und-hepatologie/
SMP, Blueprint		Internal Medicine / Haematology / Internal Medicine Oncology	A	https://www.arztakademie.at/pruefungen/oeaek-facharztpruefung/informationen-zu-den-einzelnen-sonderfaechern/innere-medizin-und-haematologie-und-intern-onkologie/
SMP, Blueprint		Internal Medicine / Infectiology	A	https://www.arztakademie.at/pruefungen/oeaek-facharztpruefung/informationen-zu-den-einzelnen-sonderfaechern/innere-medizin-und-infektiologie/
SMP, Blueprint		Internal Medicine / Intensive Care Medicine	A	https://www.arztakademie.at/pruefungen/oeaek-facharztpruefung/informationen-zu-den-einzelnen-sonderfaechern/innere-medizin-und-intensivmedizin/
SMP, Blueprint		Internal Medicine / Cardiology	A	https://www.arztakademie.at/pruefungen/oeaek-facharztpruefung/informationen-zu-den-einzelnen-sonderfaechern/innere-medizin-und-kardiologie/
SMP, Blueprint		Internal Medicine / Nephrology	A	https://www.arztakademie.at/pruefungen/oeaek-facharztpruefung/informationen-zu-den-einzelnen-sonderfaechern/innere-medizin-und-nephrologie/

SMP, Blueprint		Internal Medicine/ Rheumatology	A	https://www.arztakademie.at/pruefungen/oeaek-facharztpruefung/informationen-zu-den-einzelnen-sonderfaechern/innere-medizin-und-rheumatologie/
SMP	60	Children / Adolescent Surgery	A	https://www.arztakademie.at/pruefungen/oeaek-facharztpruefung/informationen-zu-den-einzelnen-sonderfaechern/kinder-und-jugendchirurgie/
SMP		Medical Genetics	A	https://www.arztakademie.at/pruefungen/oeaek-facharztpruefung/informationen-zu-den-einzelnen-sonderfaechern/medizinische-genetik/
SMP		Medical Performance Physiology	A	
SMP, Blueprint		Medical / Chemical Laboratory Diagnostics	A	https://www.arztakademie.at/pruefungen/oeaek-facharztpruefung/informationen-zu-den-einzelnen-sonderfaechern/medizinische-und-chemische-labordiagnostik/
SMP, Blueprint		Mouth / Jaw / Facial Surgery	A	https://www.arztakademie.at/pruefungen/oeaek-facharztpruefung/informationen-zu-den-einzelnen-sonderfaechern/mund-kiefer-und-gesichtschirurgie/
SMP		Neurobiology	A	https://www.arztakademie.at/pruefungen/oeaek-facharztpruefung/informationen-zu-den-einzelnen-sonderfaechern/neurobiologie/
SMP, Blueprint	120	Neurosurgery	A	https://www.arztakademie.at/pruefungen/oeaek-facharztpruefung/informationen-zu-den-einzelnen-sonderfaechern/neurochirurgie/
SMP, Blueprint	90	Neurology	A	https://www.arztakademie.at/pruefungen/oeaek-facharztpruefung/informationen-zu-den-einzelnen-sonderfaechern/neurologie/
SMP		Neuropathology	A	
SMP		Nuclear medicine	A	https://www.arztakademie.at/pruefungen/oeaek-facharztpruefung/informationen-zu-den-einzelnen-sonderfaechern/nuklearmedizin/
SMP, Blueprint		Orthopaedics / Orthopaedic Surgery	A	https://www.arztakademie.at/pruefungen/oeaek-facharztpruefung/informationen-zu-den-einzelnen-sonderfaechern/orthopaedie-und-orthopaedische-chirurgie/
SMP		Pathophysiology	A	https://www.arztakademie.at/pruefungen/oeaek-facharztpruefung/informationen-zu-den-einzelnen-sonderfaechern/pathophysiologie/
SMP		Pharmacology / Toxicology	A	https://www.arztakademie.at/pruefungen/oeaek-facharztpruefung/informationen-zu-den-einzelnen-sonderfaechern/pharmakologie-und-toxikologie/
SMP		Physical Med / General Rehabilitation	A	https://www.arztakademie.at/pruefungen/oeaek-facharztpruefung/informationen-zu-den-einzelnen-sonderfaechern/physikalische-medizin-und-allgemeine-rehabilitation/
SMP		Physiology	A	
SMP, Blueprint	80	Plastic / Aesthetic / Reconstructive Surgery	A	https://www.arztakademie.at/pruefungen/oeaek-facharztpruefung/informationen-zu-den-einzelnen-sonderfaechern/plastische-rekonstruktive-und-aesthetische-chirurgie/
SMP, Blueprint		Psychiatry / Psychoth. Medicine	A	https://www.arztakademie.at/pruefungen/oeaek-facharztpruefung/informationen-zu-den-einzelnen-sonderfaechern/psychiatrie/
SMP	120	Social Medicine	A	https://www.arztakademie.at/pruefungen/oeaek-facharztpruefung/informationen-zu-den-einzelnen-sonderfaechern/sozialmedizin/
SMP, Blueprint		Specific Prophylaxis / Tropical Medicine	A	https://www.arztakademie.at/pruefungen/oeaek-facharztpruefung/informationen-zu-den-einzelnen-sonderfaechern/spezifische-prophylaxe-und-tropenmedizin/
SMP, Blueprint	120	Radiotherapy / Radiooncology	A	https://www.arztakademie.at/pruefungen/oeaek-facharztpruefung/informationen-zu-den-einzelnen-sonderfaechern/strahlentherapie-und-radioonkologie/
SMP, Blueprint		Thoracic Surgery	A	https://www.arztakademie.at/pruefungen/oeaek-facharztpruefung/informationen-zu-den-einzelnen-sonderfaechern/plastische-rekonstruktive-und-aesthetische-chirurgie/
SMP, Blueprint	60	Virology	A	https://www.arztakademie.at/pruefungen/oeaek-facharztpruefung/informationen-zu-den-einzelnen-sonderfaechern/virologie/

* = in minutes
