# Supplementary material for: Assessment methods in medical specialist assessments in the DACH region – overview, critical examination and recommendations for further development
Source: GMS J Med Educ. 2019 Nov 15;36(6):Doc78. doi: 10.3205/zma001286 (PMC6905366; doi:10.3205/zma001286)
Supplement: Overview of oral assessments taking place in Germany [file JME-36-6-78-s-004.pdf]

Description	Duration*	Specialist field	Country	Source
Unstructured oral assessment	30	All medical specialist titles	Baden-Württemberg	http://www.aerztekammer-bw.de/10aerzte/30weiterbildung/40merkblaetter/10pruefung.pdf
Unstructured oral assessment	30	All medical specialist titles	Württemberg	http://www.blaek.de/
Unstructured oral assessment	30	All medical specialist titles	Bavaria	https://www.aerztekammer-berlin.de/10arzt/15_Weiterbildung/ 12WB-Informationen/Pruefungs-Flyer.pdf
Unstructured oral assessment	30	All medical specialist titles	Berlin	https://www.laekb.de/files/142FB9BFDF1/wbo_2011_konsolidierte_ Fassung.pdf
Unstructured oral assessment	30	All medical specialist titles	Brandenburg	https://www.aekhb.de/data/mediapool/AE_RE_RG_WEITERBILDUNGS-ORDNUNG.pdf
Unstructured oral assessments, sessions being taped, assessment questions delivered to the chairman beforehand.	30-60	All medical specialist titles	Bremen	http://www.aerztekammer-hamburg.org/pruefungen.html
Unstructured oral assessment	30	All medical specialist titles	Hamburg	http://www.laekh.de/images/Aerzte/Weiterbildung/WBO_2005_10.pdf
Unstructured oral assessment	30	All medical specialist titles	Hessen	http://www.aek-mv.de/upload/file/aerzte/Weiterbildung/ Weiterbildungsordnung/13_A_WBOMV.pdf
Unstructured oral assessment	30	All medical specialist titles	Mecklenburg	https://www.aekn.de/weiterbildung/pruefung/
Unstructured oral assessment	30	All medical specialist titles	Mecklenburg-Vorpommern	http://www.laek-rlp.de/downloads/wbo.pdf
Unstructured oral assessment	30	All medical specialist titles	Lower Saxony	https://www.aekno.de/downloads/archiv/2015.09.024.pdf
Unstructured oral assessment	30	All medical specialist titles	Rhineland-Palatinate	https://www.aerztekammer-saarland.de/files/155DF624FCB/WB-0001%20Merkblatt%20Aerztliche%20Weiterbildung.pdf
Unstructured oral assessment	30	All medical specialist titles	North Rhine-Westphalia	http://www.slaek.de/de/05/aufgaben/weiterbildung/pdf/ weiterbildungsordnung_neu.pdf
Unstructured oral assessment	30	All medical specialist titles	Saarland	https://www.aeksa.de/files/1465CEF5B21/WBOSA%20ab%2001.01.2015-5.%20Satzungs%C3%A4nderung-KV%2008.11.2014.pdf
Unstructured oral assessment	30	All medical specialist titles	Saxony	https://www.aeksh.de/system/files/documents/wbo_2011-05-25_idf_2016-06-22.pdf
Unstructured oral assessment	30	All medical specialist titles	Saxony Anhalt	http://www.laek-thueringen.de/wcms/ DocsID/B2B8ECED1A13D63CC1258013002CEDB2/$file/Weiterbildungsordnung %20vom%2014.%20Juli%202011%2C%20zuletzt%20ge%C3%A4ndert%20am% 2025.%20September%202014%20.pdf
Unstructured oral assessment	30	All medical specialist titles	Schleswig Holstein	http://www.aekwl.de/fileadmin/weiterbildung/WO/WO_2014_Sonderdruck_idF__2014-09-20-4.pdf

* = in minutes
